# Diabetes, Prediabetes, and the Risk of a Composite Outcome of Long-term Sickness Absence and Pre-retirement Death Due to Physical Disorders

**DOI:** 10.2188/jea.JE20220245

**Published:** 2024-03-05

**Authors:** Ami Fukunaga, Yosuke Inoue, Tohru Nakagawa, Toru Honda, Shuichiro Yamamoto, Hiroko Okazaki, Makoto Yamamoto, Toshiaki Miyamoto, Takeshi Kochi, Masafumi Eguchi, Naoki Gommori, Kenya Yamamoto, Ai Hori, Maki Konishi, Nobumi Katayama, Isamu Kabe, Seitaro Dohi, Tetsuya Mizoue

**Affiliations:** 1 Department of Epidemiology and Prevention, Center for Clinical Sciences, National Center for Global Health and Medicine, Tokyo, Japan; 2 Hitachi Health Care Center, Hitachi, Ltd., Ibaraki, Japan; 3 Mitsui Chemicals, Inc., Tokyo, Japan; 4 Yamaha Corporation, Shizuoka, Japan; 5 East Nippon Works, Nippon Steel Corporation, Chiba, Japan; 6 Furukawa Electric Co., Ltd., Tokyo, Japan; 7 East Japan Works (Keihin), JFE Steel Corporation, Kanagawa, Japan; 8 Division of Chemical Information, National Institute of Occupational Safety and Health, Kanagawa, Japan; 9 Department of Global Public Health, Faculty of Medicine, University of Tsukuba, Ibaraki, Japan; 10 Kubota Corporation, Tokyo, Japan

**Keywords:** diabetes, prediabetes, mortality, sickness absence, labor loss

## Abstract

**Background:**

Diabetes and prediabetes have been linked with morbidity or mortality from cardiovascular disease, cancer, or other physical disorders among working-age populations, but less is known about outcomes directly related to labor loss (eg, Tlong-term sickness absence [LTSA] or pre-retirement death due to physical disorders).

This prospective study aimed to examine the association of diabetes and prediabetes with the risk of a composite outcome of LTSA and pre-retirement death due to physical disorders. The present study also examined the associations of severe outcomes (LTSA or death) due to specific physical disorders or injuries/external causes in relation to diabetes and prediabetes.

**Methods:**

Data were derived from the Japan Epidemiology Collaboration on Occupational Health study. A total of 60,519 workers from 12 companies were followed for 8 years. Diabetes and prediabetes were defined based on the American Diabetes Association criteria. A Cox proportional hazards regression model was used to examine the association between diabetes/prediabetes and severe outcomes due to physical disorders or injuries/external causes.

**Results:**

The adjusted hazard ratios of severe outcomes due to all physical disorders were 1.22 (95% confidence interval [CI], 1.02–1.45) and 2.32 (95% CI, 2.04–2.64) for prediabetes and diabetes, respectively. In cause-specific analyses, an increased risk was observed for severe outcomes due to cancers, cardiovascular diseases, diseases of the musculoskeletal system, and injuries/external causes in relation to either or both diabetes and prediabetes.

**Conclusion:**

Diabetes and prediabetes were associated with an increased risk of severe outcomes due to physical disorders or injuries/external causes among Japanese workers.

## INTRODUCTION

Diabetes is the leading cause of morbidity and mortality worldwide^1^ and has been extensively studied in relation to a wide range of negative health outcomes. Epidemiological studies have shown the association between diabetes and a variety of physical disorders (eg, cardiovascular disease [CVD],^2^^,^^3^ cancer,^4^ and musculoskeletal disorder^5^) and mortality from physical disorders.^6^ A meta-analysis of 97 prospective studies showed that people with diabetes had 1.25 times higher risk of death due to cancers and 2.32 times higher risk of death due to vascular causes than those without diabetes.^6^

Additionally, a growing body of literature has focused on prediabetes, the precursor stage of diabetes, and investigated its association with the incidence of specific physical disorders (eg, CVD^7^^,^^8^ and cancer^9^) or death due to such disorders.^6^^,^^10^ For instance, a meta-analysis of 107 prospective cohort studies showed 1.15 times increased risk of CVD among those with prediabetes compared to their healthy counterparts.^7^ In relation to cancer, a meta-analysis of 16 prospective cohort studies found that prediabetes was associated with 1.15 times higher risk of cancer.^9^ These lines of evidence point to the importance of prediabetes as a condition that has a direct link to major health outcomes.

While diabetes and prediabetes have been studied in relation to physical disorders or mortality due to physical disorders among working-age population, some important issues remain to be addressed. First, less is known about the impact of diabetes or prediabetes on sickness absence (SA) or pre-retirement death caused by physical disorders. As for SA due to physical disorders, Virtanen et al^11^ showed differences in mean annual number of days in SA due to some physical disorders between those with and without diabetes using a nationwide register-based data in Sweden: musculoskeletal diseases (12.1–12.8 days/year); circulatory diseases (5.9–6.5 days/year); and diseases of the nervous system disorders (1.8–2.0 days/year). Similarly, Dray-Spira et al^12^ found a higher incidence of SA due to metabolic and circulatory causes in those with diabetes than in those without diabetes in an occupational cohort of the French National Electricity and Gas Company. Second, pre-retirement death was not considered in the above-mentioned studies investigating the association between diabetes and SA due to physical disorders.^11^^,^^12^ This is an important issue, given that considerable work loss is associated with pre-retirement death. For example, we previously showed that the disease burden of cancer due to pre-retirement death (measured in terms of the proportion of working life years lost) was two times higher than that due to long-term sickness absence (LTSA) in Japan.^13^ It is thus preferable to treat them as a composite outcome, rather than evaluating either alone. Third, it is also important to investigate the associations of such composite outcome of SA and pre-retirement death due to specific physical disorders (eg, CVD, cancer, and musculoskeletal disorder) as well as for injuries/external causes in relation to diabetes, given that previous studies indicated specific associations with SA due to such causes.^11^^,^^12^ Fourth, no previous study has investigated the risk of the composite outcome of SA and pre-retirement death due to physical disorders in relation to prediabetes. Such a study would provide additional evidence highlighting the need for the prevention targeting prediabetes to minimize the risk of severe health outcomes.

To address these issues, we prospectively examined the association of diabetes and prediabetes with a composite outcome of LTSA and pre-retirement death due to physical disorders using data from the Japan Epidemiology Collaboration on Occupational Health (J-ECOH) study.

## METHODS

### J-ECOH study

Data for the present study were derived from the J-ECOH study, which is an ongoing epidemiological study of Japanese workers across various industries (eg, electric machinery and apparatus, chemical, gas, steel, automobile, and instrument manufacturing) in Japan.^14^^,^^15^ We invited companies headquartered in the Kanto and Tokai regions of Japan via an occupational physician network (convenience sampling); the J-ECOH study mainly involves large-scale companies in Japan.

The study protocol was approved by the ethics committee of the National Center for Global Health and Medicine, Japan (approval number: NCGM-G-001140).

### Health check-up

In Japan, employers must organize annual health check-up for their employees under the Industrial Safety and Health Act. We obtained information on health check-up at each participating company, which consists of anthropometric measurements, physical examinations, laboratory examinations, and self-reported questionnaires on medical history and health-related lifestyle factors.

Body weight and height were measured using a standardized protocol (ie, participants wearing light clothes and no shoes). Blood pressure was measured in the sitting position using automatic or mercury sphygmomanometer. The levels of total cholesterol, low-density lipoprotein cholesterol, high-density lipoprotein cholesterol, and triglycerides were measured using the enzymatic method. Plasma glucose was measured using either the enzymatic or glucose oxidase peroxidative electrode method, and glycated hemoglobin (HbA1c) was measured using the latex agglutination immunoassay, high-performance liquid chromatography, or the enzymatic method.

### Assessment of diabetes and prediabetes

We identified people with diabetes or prediabetes using health check-up data (plasma glucose, HbA1c, and self-reported information on anti-diabetic treatment). According to the American Diabetes Association (ADA) criteria,^16^ diabetes was defined as random plasma glucose ≥200 mg/dL, fasting plasma glucose (FPG) ≥126 mg/dL, or HbA1c ≥6.5%. In addition, those under anti-diabetic treatment were also classified in this category. Prediabetes was defined as FPG 100–125 mg/dL or HbA1c 5.7–6.4% in those without diabetes, and normoglycemia was defined as FPG <100 mg/dL and HbA1c <5.7%.

The criteria for prediabetes by the ADA (FPG 100–<126 mg/dL or HbA1c 5.7–<6.5%)^16^ differ from the World Health Organization (WHO) (FPG 110–<126 mg/dL)^17^/International Expert Committee (IEC) (HbA1c 6.0–6.5%)^18^ criteria. Considering this heterogeneity in prediabetes criteria, we categorized prediabetes into two groups: stage I prediabetes and stage II prediabetes for additional analysis. Stage I prediabetes was defined as FPG 100–<110 mg/dL or HbA1c 5.7–<6.0% (ie, prediabetes identified only by the ADA definition), and stage II prediabetes as FPG 110–<126 mg/dL or HbA1c 6.0–<6.5% (ie, prediabetes identified by both the ADA and the WHO/IEC criteria) among those without diabetes.

### Disease registry and assessment of outcomes

Within the J-ECOH study, a set of registries was established in April 2012 to collect information on major health-related events (eg, LTSA, CVD events, and deaths). Based on the best available information, occupational physicians of the participating companies fill in standard forms and provide them to the study group. Information on the cause of LTSA was ascertained via medical certificates, which were written by attending physicians (general practitioners or specialists) and submitted by employees to their company when applying for paid SA. Information on death (eg, the cause and date of death) was based on death certificates, family confirmation, SA documents (for those who died during LTSA), and other sources.

While there is no universal definition of LTSA, it is commonly defined as SA that lasted ≥4 weeks.^19^ In the J-ECOH study, LTSA was defined as medically certified SA that lasted ≥30 consecutive days; this cutoff was determined due to multiple reasons including the availability of information by collaborating occupational physicians, the burden of data extraction and submission, and study resources.

The main outcome of this study was a composite outcome of LTSA and pre-retirement death due to physical disorders (first incidence of LTSA or death due to physical disorders). Based on the International Statistical Classification of Diseases and Related Health Problems, 10th revision (ICD-10), we defined the causes of LTSA and death. We defined the composite outcome of LTSA and death due to all physical disorders (ICD-10 codes: A00–B99, C00–D49, D50–D89, E00–E89, G00–G99, H00–H59, H60–H95, I00–I99, J00–J99, K00–K95, L00–L99, M00–M99, N00–N99, O00–O99, P00–P96, Q00–Q99, R00–R99, V00–Y99, Z00–Z99). In addition, we specifically examined severe outcomes (LTSA or death) due to cancers (ICD-10: C00–D49), CVDs (ICD-10: I00–I99), and diseases of the musculoskeletal system and connective tissues (ICD-10: M00–M99), which are the major causes of LTSA and death due to physical disorders in this cohort. We also defined the composite outcome of LTSA and death due to injuries/external causes (ie, injury, poisoning, and certain other consequences of external causes) (ICD-10: S00–T98).

### Covariates

Covariates included age (years, continuous), sex (men or women), body mass index (BMI), smoking status, hypertension, and dyslipidemia. BMI was categorized into four groups (<18.5, 18.5–24.9, 25.0–29.9, or ≥30.0 kg/m^2^). Hypertension was defined as systolic blood pressure ≥140 mm Hg, diastolic blood pressure ≥90 mm Hg, or the use of antihypertensive medication. Dyslipidemia was defined as triglyceride level ≥150 mg/dL, low-density lipoprotein cholesterol level ≥140 mg/dL, high-density lipoprotein cholesterol level <40 mg/dL, or the use of medical treatment for dyslipidemia.

### Analytic cohort

**Figure 1.**
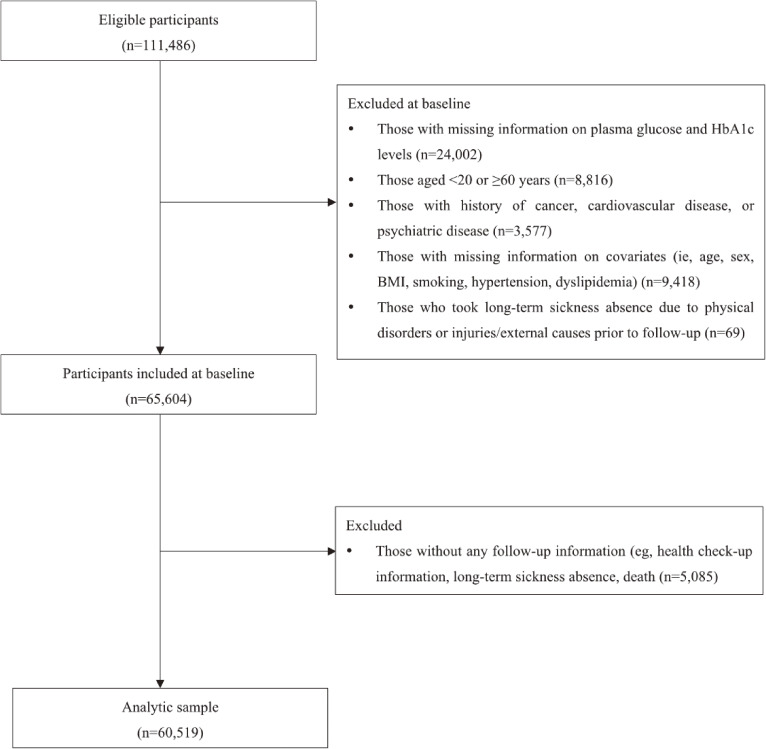
Flow chart of participant selection

For the present study, we used data from 12 participating companies that provided health check-up data in fiscal year of 2011 (if not, 2010) (10 companies) and 2014 (if not, 2013) (2 companies) (ie, the latest health check-up data available before the launch of the follow-up registry at each company). Of 111,486 eligible participants who had baseline health check-up information, we excluded: (1) those with missing information on plasma glucose and HbA1c levels (*n* = 24,002); (2) those aged younger than 20 years (the legal age of smoking) or those aged 60 years (the general retirement age) or older (*n* = 8,816); (3) those with self-reported current or past medical history of cancer, CVD, or psychiatric disease (*n* = 3,577); (4) those with missing information on selected covariates (*n* = 9,418); and (5) those who took LTSA due to physical disorders or injuries/external causes prior to follow-up and were still on LTSA when the disease registry started (*n* = 69). We then excluded those without any follow-up information (eg, health check-up information, LTSA, death) (*n* = 5,085). Consequently, 60,519 participants (51,454 men and 9,065 women) were included in subsequent analyses (Figure 1).

### Statistical analysis

The baseline characteristics of the participants were presented as means and standard deviations for continuous variables and as percentages for categorical variables. Person-years were calculated from March 31, 2012 (10 companies) or March 31, 2015 (2 companies) (ie, 1 day prior to the first day of the follow-up) to the date of the first occurrence of severe outcome due to physical disorders (LTSA or death, whichever occurred first), the date of censoring event (ie, the last participation of annual health check-up, LTSA, death due to other causes), or the end of follow-up (March 31, 2020; 3 companies ended the follow-up in 2017 or 2018). For cause-specific analysis, those with severe outcome due to other causes were censored on the date of the outcome.

Cox proportional hazards regression analysis was performed to calculate the hazard ratio (HR) and 95% confidence intervals (CIs) for severe outcomes due to all physical disorders, specific physical disorders (eg, cancers, CVDs, and diseases of the musculoskeletal system and connective tissue), or injuries/external causes in relation to the baseline diabetes/prediabetes status. Model 1 was adjusted for age, sex, BMI categories, and smoking status. Model 2 was further adjusted for hypertension and dyslipidemia. For all analyses, we treated the worksites as clusters.

To examine the robustness of the study findings, we conducted a series of sensitivity analyses. First, we conducted stratified analysis by age categories (<45 or ≥45 years old), sex (men or women), BMI categories (<25 or ≥25 kg/m^2^), and hypertension status (without or with hypertension). Second, we conducted an analysis where only LTSA was the outcome measure.

All statistical analyses were performed using SAS version 9.4 (SAS Institute, Cary, NC, USA). Statistical significance was set at a *P*-value of <0.05 (two-tailed).

## RESULTS

During the maximum 8-year follow-up period (mean: 6.1 years), 1,570 participants had severe outcomes (LTSA or death) due to physical disorders or injuries/external causes (1,312 for physical disorders and 258 for injuries/external causes). Of the 1,312 severe outcomes due to physical disorders, 423 (32.2%) were due to cancers, 266 (20.3%) were due to CVDs, and 216 (16.5%) were due to diseases of the musculoskeletal system and connective tissue (eTable 1). Major injuries/external causes included injuries to the knee and lower leg (33.7%), ankle and foot (11.2%), shoulder and upper arm (10.5%), and abdomen, lower back, lumbar spine, and pelvis (8.1%) (eTable 1).

Table 1 shows the characteristics of the study participants according to their baseline diabetes status. In this cohort, 49.9%, 43.2%, and 6.9% of the participants were those with normoglycemia, prediabetes, and diabetes, respectively. Compared with those with normoglycemia, those with prediabetes and diabetes were more likely to be older, men, overweight/obese, current smokers, and have hypertension and dyslipidemia.

**Table 1.** Baseline characteristics of study participants, the J-ECOH study, Japan (*n* = 60,519)

Characteristics	All participants (*n* = 60,519)	Diabetes status		

		Normoglycemia^a^ (*n* = 30,217)	Prediabetes^b^ (*n* = 26,115)	Diabetes^c^ (*n* = 4,187)
Age, mean [SD]	43.7 [9.4]	40.8 [9.5]	46.0 [8.5]	49.9 [7.5]
Sex, men, *n* (%)	51,454 (85.0)	24,522 (81.2)	23,012 (88.1)	3,920 (93.6)
BMI categories, *n* (%)				
<18.5 kg/m^2^	2,928 (4.8)	2,061 (6.8)	814 (3.1)	53 (1.3)
18.5–24.9 kg/m^2^	40,636 (67.2)	22,437 (74.3)	16,431 (62.9)	1,768 (42.2)
25.0–29.9 kg/m^2^	14,060 (23.2)	5,069 (16.8)	7,371 (28.2)	1,620 (38.7)
≥30.0 kg/m^2^	2,895 (4.8)	650 (2.2)	1,499 (5.7)	746 (17.8)
Smoking status, *n* (%)				
Never	27,034 (44.7)	15,181 (50.2)	10,538 (40.4)	1,315 (31.4)
Former	12,881 (21.3)	5,541 (18.3)	6,262 (24.0)	1,078 (25.8)
Current	20,604 (34.1)	9,495 (31.4)	9,315 (35.7)	1,794 (42.9)
Hypertension, *n* (%)	11,407 (18.9)	3,708 (12.3)	5,786 (22.2)	1,913 (45.7)
Dyslipidemia, *n* (%)	26,821 (44.3)	10,242 (33.9)	13,720 (52.5)	2,859 (68.3)

BMI, body mass index; FPG, fasting plasma glucose; HbA1c, glycated hemoglobin; SD, standard deviation.

^a^Normoglycemia: FPG <100 mg/dL and HbA1c <5.7%.

^b^Prediabetes: FPG 100–125 mg/dL or HbA1c 5.7–6.4%.

^c^Diabetes: random plasma glucose ≥200 mg/dL, FPG ≥126 mg/dL, or HbA1c ≥6.5%, or the use of anti-diabetic treatment.

Table 2 shows the association between diabetes/prediabetes and severe outcomes (LTSA or death) due to all physical disorders, specific physical disorders, and injuries/external causes. Compared with those with normoglycemia, HRs of severe outcomes due to all physical disorders were 1.22 (95% CI, 1.02–1.45) and 2.32 (95% CI, 2.04–2.64) for prediabetes and diabetes, respectively. The risk was more pronounced in those with stage II prediabetes (HR 1.38; 95% CI, 1.15–1.65) than in those with stage I prediabetes (HR 1.16; 95% CI, 0.96–1.39).

**Table 2.** Hazard ratios and 95% confidence intervals for the composite outcome of LTSA and death due to physical disorders or injuries/external causes according to baseline diabetes status

	Normoglycemia^a^ (*n* = 30,217)	Prediabetes^b^ (*n* = 26,115)	Diabetes^c^ (*n* = 4,187)	Prediabetes^b^ (*n* = 26,115)	

				Stage I prediabetes^d^ (*n* = 18,962)	Stage II prediabetes^e^ (*n* = 7,153)
Person-years	187,234	161,302	23,577	118,099	43,205
**1. LTSA or death due to all physical disorders**					
Number of events	468	619	225	407	212
Model 1	1.00 (reference)	**1.24 (1.04–1.47)**	**2.48 (2.20–2.81)**	1.17 (0.97–1.41)	**1.42 (1.19–1.70)**
Model 2	1.00 (reference)	**1.22 (1.02–1.45)**	**2.32 (2.04–2.64)**	1.16 (0.96–1.39)	**1.38 (1.15–1.65)**
**1-1. LTSA or death due to cancers**					
Number of events	158	219	46	141	78
Model 1	1.00 (reference)	1.18 (0.98–1.42)	1.32 (0.98–1.79)	1.11 (0.89–1.38)	**1.36 (1.12–1.66)**
Model 2	1.00 (reference)	1.17 (0.97–1.41)	1.27 (0.98–1.63)	1.10 (0.88–1.38)	**1.34 (1.08–1.65)**
**1-2. LTSA or death due to cardiovascular diseases**					
Number of events	77	132	57	80	52
Model 1	1.00 (reference)	1.22 (0.80–1.86)	**2.26 (1.37–3.70)**	1.12 (0.75–1.70)	1.44 (0.83–2.51)
Model 2	1.00 (reference)	1.15 (0.76–1.74)	**1.93 (1.22–3.05)**	1.07 (0.72–1.61)	1.31 (0.76–2.27)
**1-3. LTSA or death due to diseases of the ** **musculoskeletal system and connective tissue**					
Number of events	67	117	32	85	32
Model 1	1.00 (reference)	**1.67 (1.20–2.32)**	**2.49 (1.43–4.35)**	**1.73 (1.21–2.47)**	**1.51 (1.09–2.09)**
Model 2	1.00 (reference)	**1.66 (1.21–2.28)**	**2.37 (1.37–4.10)**	**1.72 (1.22–2.42)**	**1.48 (1.07–2.04)**
**2. LTSA or death due to injuries/external causes**					
Number of events	106	123	29	81	42
Model 1	1.00 (reference)	1.14 (0.77–1.70)	**1.58 (1.13–2.20)**	1.07 (0.64–1.79)	**1.34 (1.05–1.71)**
Model 2	1.00 (reference)	1.14 (0.74–1.74)	**1.51 (1.10–2.08)**	1.07 (0.62–1.84)	**1.32 (1.02–1.71)**

FPG, fasting plasma glucose; HbA1c, glycated hemoglobin; LTSA, long-term sickness absence.

^a^Normoglycemia: FPG <100 mg/dL and HbA1c <5.7%.

^b^Prediabetes: FPG 100–125 mg/dL or HbA1c 5.7–6.4%.

^c^Diabetes: random plasma glucose ≥200 mg/dL, FPG ≥126 mg/dL, or HbA1c ≥6.5%, or the use of anti-diabetic treatment.

^d^Stage I prediabetes: FPG 100–<110 mg/dL or HbA1c 5.7–<6.0%.

^e^Stage II prediabetes: FPG 110–<126 mg/dL or HbA1c 6.0–<6.5%.

Model 1, adjusted for age (years, continuous), sex (men or women), BMI categories (<18.5, 18.5–24.9, 25.0–29.9, or ≥30.0 kg/m^2^), and smoking status (never, former, or current).

Model 2, further adjusted for hypertension (yes or no) and dyslipidemia (yes or no).

For specific physical disorders, the risk of severe outcomes due to CVDs was significantly increased in those with diabetes (HR 2.26; 95% CI, 1.37–3.70), but not in those with prediabetes (HR 1.15; 95% CI, 0.76–1.74). We observed an increasing trend in the risk of a severe outcomes due to cancers in those with prediabetes (HR 1.17; 95% CI, 0.97–1.41) and diabetes (HR 1.27; 95% CI, 0.98–1.63) at statistically non-significant level while the risk was significantly higher in those with stage II prediabetes (HR 1.34; 95% CI, 1.08–1.65). The risk of severe outcomes due to diseases of the musculoskeletal system and connective tissues was significantly higher among those with diabetes (HR 2.37; 95% CI, 1.37–4.10) and prediabetes (HR 1.66; 95% CI, 1.21–2.28). Additionally, diabetes (HR 1.51; 95% CI, 1.10–2.08) as well as stage II prediabetes (HR 1.32; 95% CI, 1.02–1.71) were both associated with an increased risk of severe outcomes due to injuries/external causes.

In stratified analysis, the associations were similar across different groups by age categories, sex, BMI categories, and hypertension status (eTable 2). In the sensitivity analysis where we accounted only LTSA as the outcome, the associations were virtually the same as those of the composite outcome of LTSA and death (eTable 3).

## DISCUSSION

In this large-scale prospective study among workers in Japan, diabetes and prediabetes were significantly associated with a higher risk of severe outcomes (LTSA or death) due to all physical disorders. In cause-specific analysis, diabetes was associated with the risk of severe outcomes due to CVDs, diseases of the musculoskeletal system and connective tissues, and injuries/external causes, whereas prediabetes was associated with the risk of severe outcomes due to cancers, diseases of the musculoskeletal system and connective tissues, and injuries/external causes.

Epidemiological studies have shown the association between diabetes and SA^11^^,^^12^ or mortality due to physical disorders.^6^^,^^10^ The present study extends this evidence by showing the association between diabetes and the composite outcome of LTSA and pre-retirement death due to physical disorders. The increased risk of severe outcomes due to all physical disorders among those with prediabetes corroborated with studies showing an increased risk of mortality due to physical disorders among those with prediabetes.^6^^–^^8^^,^^10^ Importantly, we found that the risk was pronounced only in stage II prediabetes. The results of the present study indicate that both diabetes and prediabetes were associated with an increased risk of severe outcomes due to physical disorders in a working population. It is also of note that the results did not differ markedly across different groups of participants stratified by age categories, sex, BMI categories, and hypertension status.

In our cause-specific analyses, diabetes but not prediabetes was associated with severe outcomes due to CVDs. The finding of an increased risk of the outcomes among those with diabetes was in line with previous studies showing a significant association between diabetes and an increased risk of LTSA^11^^,^^12^ or mortality^6^ due to CVDs. A French cohort study showed a higher incidence rate of LTSA (SA that lasted >28 days) due to circulatory causes among those with self-reported diabetes than among those without diabetes (14.2 vs 4.8 per 1,000 person-years, *P* < 0.001).^12^ We observed a slightly increased, albeit statistically non-significant, risk of severe outcomes due to CVDs among those with prediabetes (HR 1.15; 95% CI, 0.76–1.74). This point estimate is in accordance with meta-analyses showing a small yet significantly increased risk of composite CVD events in relation to prediabetes.^7^^,^^8^ For example, a recent meta-analysis of 107 observational studies by Cai et al^7^ documented that prediabetes (defined as impaired fasting glucose, impaired glucose tolerance [IGT], or elevated HbA1c) was associated with 1.15 times increased risk of CVD. Further investigation is needed to examine the association between hyperglycemia and severe CVD outcomes.

While we observed an increased risk of severe outcomes due to cancers among those with diabetes or prediabetes, this association was found to be statistically significant only in stage II prediabetes (HR 1.34; 95% CI, 1.09–1.63). This association is consistent with previous studies showing increased cancer mortality associated with diabetes.^6^^,^^10^ In relation to prediabetes, a meta-analysis revealed that impaired FPG levels (defined as FPG levels ≥100 mg/dL without a history of diabetes) were associated with an increased risk of mortality due to cancers (HR 1.13; 95% CI, 1.06–1.20).^6^ The link between hyperglycemia and severe outcomes due to cancers is plausible, as hyperglycemia could promote tumor progression through a variety of biological mechanisms, such as tumor cell proliferation, migration, and invasion.^20^

We found a significant association between diabetes and prediabetes with severe outcomes (ie, LTSA) due to diseases of the musculoskeletal system and connective tissue (HR 1.93 and HR 1.66 for diabetes and prediabetes, respectively), which mainly consisted of spondylopathies (32%) (ICD-10: M45–M49), dorsopathies (32%) (ICD-10: M50–M54), and osteoarthritis (12%) (ICD-10: M15–M19). One Swedish cohort study reported that the mean annual number of days in SA due to musculoskeletal disorders (ICD-10: M00–M99) was longer among people with diabetes than among those without diabetes (12.1–12.8 days/year).^11^ The present study not only confirmed the association, but also extended the findings by showing an increased risk among those with prediabetes (both stage I and stage II prediabetes). While the mechanisms underlying the link between hyperglycemia and diseases of the musculoskeletal system and connective tissue remain uncertain, some potential explanations, such as musculoskeletal disorders as a consequence of diabetes complications, shared pathological mechanisms with microvascular complications, and accumulation of advanced glycosylation end products (AGEs)^21^^,^^22^ have been suggested. For example, the accumulation of AGEs due to chronic hyperglycemia can change the structure and function of a wide range of proteins, including extracellular proteins such as collagen, affecting the material properties and quality of the bone and joints.^21^^,^^22^

Diabetes and prediabetes in the advanced stage (stage II prediabetes) were both associated with a significant increase in the risk of the composite outcome of LTSA and pre-retirement death due to injuries/external causes, which mainly consisted of injuries. Similarly, a previous study showed that the mean annual number of days in SA due to injuries (ICD-10: S00–T35, T66–T78, and T79) was longer among those with diabetes (1.0–1.2 days/year) than those without diabetes.^11^ A meta-analysis of 97 studies demonstrated a link between diabetes and mortality due to external causes (HR 1.36).^6^ Symptoms or complications of diabetes (eg, extreme fatigue, impaired vision, and tingling or numbness in the hands or feet^23^) may increase the risk of injuries due to accidents in daily life.

The present study has several strengths, which include the use of data from a large-scale occupational cohort, definition of prediabetes by accounting for the heterogeneity in its ADA and WHO/IEC criteria, and consideration of the composite outcome of LTSA and death using medically certified information. However, our study has several limitations. First, we did not have data on SA that lasted for less than 30 consecutive days. Second, we could not rule out the possibility that residual and unmeasured confounding might have existed. For example, work-related stress may increase the risk of both diabetes and outcomes related to physical disorders via physiological stress response. Third, due to the lack of data from the oral glucose tolerance test, we were unable to define diabetes status based on postprandial glucose levels (eg, IGT). Finally, our study population does not represent the working population in Japan due to the sampling method (convenience sampling); thus, generalization of the findings should be made with caution.

In conclusion, diabetes and prediabetes were associated with a higher risk of the composite outcome of LTSA and pre-retirement death due to all physical disorders, cancers, CVDs, diseases of the musculoskeletal system and connective tissue, and injuries/external causes among a Japanese working population. Our findings suggest the importance of the prevention for not only diabetes but also prediabetes to reduce labor loss associated with physical disorders in working populations.

## SUPPLEMENTARY MATERIAL

eTable 1. Specific causes of LTSA and death due to physical disorders or injuries/external causes (*n* = 1,570)

eTable 2. Hazard ratios and 95% confidence intervals for the composite outcome of LTSA and death due to all physical disorders according to baseline diabetes status, stratified by age categories, sex, BMI categories, or hypertension status

eTable 3. Hazard ratios and 95% confidence intervals for LTSA due to physical disorders or injuries/external causes according to baseline diabetes status, the J-ECOH study, Japan
