# Supplementary material for: Diabetes, Prediabetes, and the Risk of a Composite Outcome of Long-term Sickness Absence and Pre-retirement Death Due to Physical Disorders
Source: J Epidemiol. 2024 Mar 5;34(3):105–11. doi: 10.2188/jea.JE20220245 (PMC10853045; doi:10.2188/jea.JE20220245)
Supplement: Supplementary file 1 [file je-34-105-s001.pdf]

eTable 1. Specific causes of LTSA and death due to physical disorders or injuries/external causes (*n*=1,570)

Description	ICD-10 code	LTSA only	Death only	LTSA and death
**Physical disorders (*n*=1,312)**				
Cancers	C00–D49	319	25	79
Cardiovascular diseases	I00–I99	201	60	5
Diseases of the musculoskeletal system and connective tissue	M00–M99	215	0	1
Diseases of the digestive system	K00–K95	70	7	2
Diseases of the nervous system	G00–G99	64	1	2
External causes of morbidity	V00–Y99	0	38	0
Endocrine, nutritional and metabolic diseases	E00–E89	33	0	0
Pregnancy, childbirth and the puerperium	O00–O99	31	0	0
Diseases of the respiratory system	J00–J99	23	4	1
Diseases of the genitourinary system	N00–N99	23	2	0
Diseases of the eye and adnexa	H00–H59	23	0	0
Certain infectious and parasitic diseases	A00–B99	20	2	0
Diseases of the skin and subcutaneous tissue	L00–L99	13	0	0
Diseases of the ear and mastoid process	H60–H95	11	0	0
Symptoms, signs and abnormal clinical and laboratory findings, not elsewhere classified	R00–R99	20	4	0
Diseases of the blood and blood-forming organs and certain disorders involving the immune mechanism	D50–D89	5	0	0
Factors influencing health status and contact with health services	Z00–Z99	5	0	0
Congenital malformations, deformations and chromosomal abnormalities	Q00–Q99	3	0	0

Certain conditions originating in the perinatal period	P00–P96	0	0	0
**Injuries/external causes (*n*=258)**				
Injuries to the knee and lower leg	S80–S89	87	0	0
Injuries to the ankle and foot	S90–S99	29	0	0
Injuries to the shoulder and upper arm	S40–S49	27	0	0
Injuries to the abdomen, lower back, lumbar spine and pelvis	S30–S39	21	0	0
Injuries to the head	S00–S09	18	2	0
Injuries to the neck	S10–S19	13	0	0
Injuries to the thorax	S20–S29	13	0	0
Injuries to the hip and thigh	S70–S79	12	0	0
Injuries to the elbow and forearm	S50–S59	11	0	0
Injuries to the wrist and hand	S60–S69	8	0	0
Other injuries/external causes		17	0	0

ICD-10, International Classification of Diseases, 10th revision; LTSA, long-term sickness absence.

eTable 2. Hazard ratios and 95% confidence intervals for the composite outcome of LTSA and death due to all physical disorders according to baseline diabetes status, stratified by age categories, sex, BMI categories, or hypertension status

	**Normoglycemia**	**Prediabetes**	**Diabetes**
**Age categories**			
<45 years old (*n*=31,441)			
Person-years	124,492	73,425	6,476
Number of events/subjects	229/19,494	188/10,962	53/985
Model 1	1.00 (reference)	**1.40 (1.07–1.84)**	**3.69 (2.75–4.95)**
Model 2	1.00 (reference)	**1.34 (1.01–1.79)**	**3.22 (2.32–4.48)**
≥45 years old (*n*=29,078)			
Person-years	62,741	87,878	17,101
Number of events/subjects	239/10,723	431/15,153	172/3,202
Model 1	1.00 (reference)	**1.26 (1.06–1.49)**	**2.42 (1.98–2.95)**
Model 2	1.00 (reference)	**1.22 (1.03–1.46)**	**2.23 (1.80–2.77)**
**Sex**			
Men (*n*=51,454)			
Person-years	153,154	143,277	22,122
Number of events/subjects	320/24,522	528/23,012	204/3,920
Model 1	1.00 (reference)	**1.33 (1.07–1.66)**	**2.52 (2.20–2.89)**
Model 2	1.00 (reference)	**1.32 (1.05–1.64)**	**2.39 (2.06–2.78)**
Women (*n*=9,065)			
Person-years	34,080	18,026	1,454
Number of events/subjects	148/5,695	91/3,103	21/267
Model 1	1.00 (reference)	0.97 (0.75–1.26)	**2.14 (1.33–3.45)**
Model 2	1.00 (reference)	0.91 (0.69–1.20)	1.71 (0.99–2.94)
**BMI categories**			
<25.0 kg/m^2 (*n*=43,564)			
Person-years	151,976	106,333	9,958

Number of events/subjects	352/24,498	387/17,245	85/1,821
Model 1	1.00 (reference)	**1.31 (1.15–1.50)**	**2.55 (2.12–3.01)**
Model 2	1.00 (reference)	**1.29 (1.13–1.48)**	**2.36 (1.94–2.87)**
≥25.0 kg/m^2 (*n*=16,955)			
Person-years	35,257	54,971	13,619
Number of events/subjects	116/5,719	232/8,870	140/2,366
Model 1	1.00 (reference)	1.09 (0.83–1.44)	**2.38 (1.89–3.00)**
Model 2	1.00 (reference)	1.06 (0.80–1.40)	**2.19 (1.74–2.76)**
**Hypertension status**			
Without hypertension (*n*=49,112)			
Person-years	166,162	128,472	13,238
Number of events/subjects	375/26,509	434/20,329	112/13,238
Model 1	1.00 (reference)	**1.23 (1.04–1.46)**	**2.49 (2.12–2.94)**
Model 2	1.00 (reference)	**1.26 (1.06–1.50)**	**2.71 (2.25–3.26)**
With hypertension (*n*=11,407)			
Person-years	21,072	32,831	10,338
Number of events/subjects	93/3,708	185/5,786	113/1,913
Model 1	1.00 (reference)	1.18 (0.88–1.58)	**2.15 (1.61–2.88)**
Model 2	1.00 (reference)	1.19 (0.88–1.61)	**2.21 (1.62–3.01)**

BMI, body mass index; HbA1c, glycated hemoglobin; LTSA, long-term sickness absence.

Model 1, adjusted for age (years, continuous), sex (men or women), BMI categories (<18.5, 18.5–24.9, 25.0–29.9, or ≥30.0 kg/m^2), and smoking status (never, former, or current).

Model 2, further adjusted for hypertension (yes or no) and dyslipidemia (yes or no).

Each factor for stratified analysis was not adjusted in each model.

eTable 3. Hazard ratios and 95% confidence intervals for LTSA due to physical disorders or injuries/external causes according to baseline diabetes status, the J-ECOH study, Japan

	**Normoglycemia [a] (*n*=30,217)**	**Prediabetes [b] (*n*=26,115)**	**Diabetes [c] (*n*=4,187)**	**Prediabetes [b] (*n*=26,115)**	
				**Stage I prediabetes [d] (*n*=18,962)**	**Stage II prediabetes [e] (*n*=7,153)**
Person-years	187,228	161,302	23,568	118,097	43,204
**1. LTSA due to all physical disorders**					
Number of events	419	563	191	368	195
Model 1	1.00 (reference)	**1.27 (1.06–1.51)**	**2.40 (2.11–2.73)**	1.19 (0.98–1.45)	**1.48 (1.26–1.74)**
Model 2	1.00 (reference)	**1.24 (1.04–1.48)**	**2.24 (1.95–2.58)**	1.17 (0.96–1.43)	**1.43 (1.21–1.68)**
**1-1. LTSA due to cancers**					
Number of events	151	205	42	131	74
Model 1	1.00 (reference)	1.16 (0.96–1.39)	1.26 (0.86–1.85)	1.08 (0.86–1.36)	**1.35 (1.12–1.63)**
Model 2	1.00 (reference)	1.14 (0.95–1.37)	1.21 (0.87–1.70)	1.07 (0.86–1.34)	**1.32 (1.09–1.60)**
**1-2. LTSA due to cardiovascular diseases**					
Number of events	59	109	38	64	45
Model 1	1.00 (reference)	1.31 (0.90–1.92)	**1.97 (1.08–3.60)**	1.18 (0.79–1.74)	1.63 (0.99–2.69)
Model 2	1.00 (reference)	1.23 (0.85–1.77)	1.68 (0.98–2.89)	1.12 (0.76–1.64)	1.48 (0.90–2.43)
**1-3. LTSA due to diseases of the musculoskeletal system and connective tissue**					
Number of events	67	117	32	85	32

Model 1	1.00 (reference)	**1.67 (1.20–2.32)**	**2.49 (1.43–4.35)**	**1.73 (1.21–2.47)**	**1.51 (1.09–2.09)**
Model 2	1.00 (reference)	**1.66 (1.21–2.28)**	**2.37 (1.37–4.10)**	**1.72 (1.22–2.42)**	**1.48 (1.07–2.04)**
**2. LTSA due to injuries/external causes**					
Number of events	106	122	28	80	42
Model 1	1.00 (reference)	1.13 (0.76–1.67)	**1.51 (1.10–2.09)**	1.06 (0.64–1.76)	**1.33 (1.05–1.70)**
Model 2	1.00 (reference)	1.12 (0.74–1.71)	**1.45 (1.07–1.96)**	1.06 (0.62–1.80)	**1.32 (1.02–1.70)**

BMI, body mass index; FPG, fasting plasma glucose; HbA1c, glycated hemoglobin; LTSA, long-term sickness absence.

[a] Normoglycemia: FPG <100 mg/dL and HbA1c <5.7%.

[b] Prediabetes: FPG 100–125 mg/dL or HbA1c 5.7–6.4%.

[c] Diabetes: random plasma glucose ≥200 mg/dL, FPG ≥126 mg/dL, or HbA1c ≥6.5%, or the use of anti-diabetic treatment.

[d] Stage I prediabetes: FPG 100–<110 mg/dL or HbA1c 5.7–<6.0%.

[e] Stage II prediabetes: FPG 110–<126 mg/dL or HbA1c 6.0–<6.5%.

Model 1, adjusted for age (years, continuous), sex (men or women), BMI categories (<18.5, 18.5–24.9, 25.0–29.9, or ≥30.0 kg/m^2), and smoking status (never, former, or current).

Model 2, further adjusted for hypertension (yes or no) and dyslipidemia (yes or no).
